# Minimally invasive plate osteosynthesis (MIPO) for scapular fractures

**DOI:** 10.1007/s00064-023-00819-5

**Published:** 2023-08-18

**Authors:** B. J. M. van de Wall, R. J. Hoepelman, C. Michelitsch, N. Diwersi, C. Sommer, R. Babst, F. J. P. Beeres

**Affiliations:** 1 Klinik für Orthopädie und Unfallchirurgie, Luzerner Kantonsspital, Spitalstrasse, 6000 Luzern, Switzerland; 2 Department of Surgery, University Medical Center Utrecht, Utrecht, The Netherlands; 3 Unfallchirurgie, Kantonsspital Graubünden, Chur, Switzerland; 4 Klinik für Chirurgie, Kantonsspital Obwalden, Sarnen, Switzerland; 5 University of Luzern, Luzern, Switzerland

**Keywords:** Minimally invasive surgical procedures, MIPO, Scapula fracture, Deltoid sparing, Minimal-invasive Osteosynthese, MIPO, Skapulafrakturen, Delta erhaltend

## Abstract

**Objective:**

Presentation of a minimally invasive surgical approach for the treatment of scapular fractures and the clinical outcome using this technique.

**Indications:**

Displaced extra-articular fractures of the scapula body and glenoid neck (AO 14B and 14F) and simple intra-articular fractures of the glenoid.

**Contraindications:**

Complex intra-articular fractures and isolated fractures of the coracoid base.

**Surgical technique:**

Make a straight or slightly curved incision along the lateral margin of the scapula leaving the deltoid fascia intact. Identify the interval between the teres minor muscle and infraspinatus to visualize the lateral column, whilst retracting the deltoid to visualize the glenoid neck. Reduce and align the fracture using direct and indirect reduction tools. A second window on the medial border of the scapula can be made to aid reduction and/or to augment stability. Small (2.0–2.7 mm) plates in a 90° configuration on the lateral border and, if required, on the medial border are used. Intra-operative imaging confirms adequate reduction and extra-articular screw placement.

**Postoperative management:**

Direct postoperative free functional nonweight-bearing rehabilitation limited to 90° abduction for the first 6 weeks. Sling for comfort. Free range of motion and permissive weight-bearing after 6 weeks.

**Results:**

We collected data from 35 patients treated with minimally invasive plate osteosynthesis (MIPO) between 2011 and 2021. Average age was 53 ± 15.1 years (range 21–71 years); 17 had a type B and 18 a type F fracture according the AO classification. All patients suffered concomitant injuries of which thoracic (*n* = 33) and upper extremity (*n* = 25) injuries were most common. Double plating of the lateral border (*n* = 30) was most commonly performed as described in the surgical technique section. One patient underwent an additional osteosynthesis 3 months after initial surgery due to pain and lack of radiological signs of healing of a fracture extension into the spine of the scapula. In the same patient, the plate on the spine of scapula was later removed due to plate irritation. In 2 patients postoperative images showed a screw protruding into the glenohumeral joint requiring revision surgery. After standardisation of intra-operative imaging following these two cases, intra-articular screw placement did not occur anymore. No patient suffered from iatrogenic nerve injury and none developed a wound infection.

## Abstract (de)

**Operationsziel:**

Präsentation des minimal-invasiven Zugangs zur Versorgung von Skapulafrakturen samt klinischem Ausgang.

**Indikationen:**

Dislozierte, extraartikuläre Frakturen des Skapulablatts sowie des Collum scapulae (AO 148 und 14F) sowie einfache intraartikuläre Frakturen des Glenoids.

**Kontraindikationen:**

Komplexe intraartikuläre Frakturen und isolierte Frakturen der Basis des Processus coracoideus.

**Operationstechnik:**

Setzen einer geraden oder leicht gebogenen Inzision entlang des lateralen Rands der Skapula unter Intaktlassen der Deltafaszie. Identifikation des Intervalls zwischen M. teres minor und M. infraspinatus zur Visualisierung des lateralen Skapulablatts. Der Deltamuskel wird dabei nach kranial weggehalten, um den Skapulahals zu visualisieren. Frakturreposition unter Verwendung von direkten und indirekten Repositionsinstrumenten. Ein zweites Fenster über der Facies medialis der Skapula kann dabei zur besseren Visualisierung oder Stabilisierung eröffnet werden. Verwendung kleiner (2,0–2,7 mm), im 90°-Winkel zueinander angeordneter Platten entlang der lateralen Begrenzung der Skapula oder – sofern nötig – medialseitig. Die intraoperative Kontrolle mittels Bildwandler bestätigt die korrekte Reposition und Lage des Osteosynthesematerials.

**Weiterbehandlung:**

Sofortige postoperative funktionelle Behandlung ohne Belastung bis zur Horizontalen während der ersten 6 Wochen. Armschlinge zum Komfort. Nach 6 Wochen freie Beweglichkeit und Belastung.

**Ergebnisse:**

Zwischen 2011 und 2021 behandelten wir 35 Patienten mit der MIPO(minimal-invasive Plattenosteosynthese)-Technik, welche im Mittel über 8 Monate (Bandbreite von 3 bis 31 Monate) nachbehandelt wurden. Das Durchschnittsalter belief sich auf 53 ± 15,1 Jahre (Bandbreite 21 bis 71); 17 Patienten wiesen eine B‑Fraktur, 18 eine F‑Fraktur nach AO(Arbeitsgemeinschaft für Osteosynthesefragen)-Klassifikation auf. In der Mehrzahl der Fälle (*n* = 30) erfolgte eine laterale Doppelplattenosteosynthese wie unter der Rubrik Operationstechnik beschrieben. Ein Patient wurde 3 Monate nach initialer Osteosynthese erneut operiert aufgrund von Schmerzen und fehlender radiologischer Konsolidation eines Frakturausläufers in die Spina scapulae. Die daraufhin eingebrachte Platte auf der Spina wurde später aufgrund von störendem Osteosynthesematerial wieder entfernt. Bei 2 Patienten erfolgte eine Revision aufgrund einer intraartikulär im Glenohumeralgelenk liegenden Schraube. Keiner der Patienten zeigte iatrogene Nervenläsionen oder Wundinfekte.

## Introductory remarks

Scapula fractures typically result from high-energy trauma and are often associated with other thoracic or upper extremity injures. Non- or minimally displaced fractures can be treated conservatively; however, malalignment of the glenoid neck (a sharpened glenopolar angle, GPA) and intra-articular malunion can lead to impingement, rotator cuff dysfunction, osteoarthritis and, consequently, poor functional outcome [1, 2, 3]. The scapula functions as a dynamic stabilizer for the humerus–shoulder complex and therefore malunion induces chronic loss of endurance, strength and muscle reaction capacity

Over the last few decades, open reduction and internal fixation according to AO principles is increasingly applied to restore the GPA, articular congruency and alignment. Cole et al. described a well differentiated assessment algorithm to determine when surgery is indicated [1].

The most common approaches for the treatment of scapula fractures are the Judet and the modified Judet approach, first described in 1964 [2]. The main advantage of the Judet approach is complete exposure of the posterior aspect of the scapula body. However, this requires a large skin incision and extensive muscular disruption by release of the Infraspinatus as well as the deltoid muscle. Furthermore, the large reflected muscle flap of the infraspinatus impedes articular visualisation and can create tension on the suprascapular nerve. The modified Judet approach uses the same incision as its traditional counterpart; however, it uses windows between the infraspinatus and teres minor to approach the fracture (instead of release of the deltoid muscle and infraspinatus). Cole et al. showed that this less invasive approach only reduces the view on the lateral column by 9% [3]. Nevertheless, it still requires an extensive cutaneous incision with the inherent risk of complications (e.g. seroma, cutaneous nerve injury, increased risk of superficial infection and aesthetic concerns).

Gauger and Cole described a minimal invasive approach working through soft tissue windows in 2011 [3]. Their outcomes were promising and extended the indication for surgical management in their and our practise. In our institute the technique has been applied for approximately 10 years.

The aim of the current paper is to enhance awareness of this approach and present our clinical experience with this technique.

## Surgical principles and objective

The minimally invasive plate osteosynthesis of scapula fractures allows for adequate fracture reduction through soft tissue windows without need to release any muscles including the posterior portion of the deltoid. Different soft tissue windows (over the lateral and medial border, as well as over the spina scapulae) are used to approach the scapula.

### Advantages

- Minimal disruption of subcutaneous tissue and posterior scapula musculature
- Potentially reduced risk of suprascapular and deltoid nerve injury due to deltoid muscle sparing approach
- Improved cosmetic outcomes

### Disadvantages

- Limited vision of the surgical field, especially if sufficient muscle relaxation has not been acquired
- Technically demanding
- Limited control of fracture fragments

### Indications

The treatment decision should be based not only on the fracture pattern but also on the needs of the individual patients. The following indications according to Cole et al. may serve as a guideline for the application [1]:

- Glenopolar angle < 22°
- > 20 mm medial–lateral displacement (MLD) of the glenohumeral part
- Angular deformity > 45°or > 30° in combination with MLD > 15 mm
- Intra-articular step-off > 4 mm
- > 10 mm MLD in combination with additional disruption of the superior shoulder suspensory complex.

### Contraindications

- Complex intra-articular fractures
- Fracture of the scapula process (AO 14A)

### Patient information

- General surgical risks
- Implant-related complications (e.g. loss of reduction, screw perforation)
- Injury to the nerves (suprascapular and axillary)
- Implant removal

### Preoperative work-up

- Clinical examination especially of motor and sensory function of the brachial plexus (including axillary and suprascapular nerve)
- A preoperative computed tomography (CT) scan including three-dimensional (3D) reconstructions is strongly recommended for fracture assessment and adequate planning

### Instruments

- Standard surgical instruments for soft tissue procedures and osteosynthesis
- Large and small pointed and blunt reduction forceps
- 2.0, 2.4, 2.7 mm LCP plates and screws, K-wires (1.2 and 1.6 mm)
- Push–pull instruments (ball spike, bone hook, Schanz screws)
- Minidistractor or minimally invasive (MI) reduction tools

### Anaesthesia and positioning

**Fig. 1:**
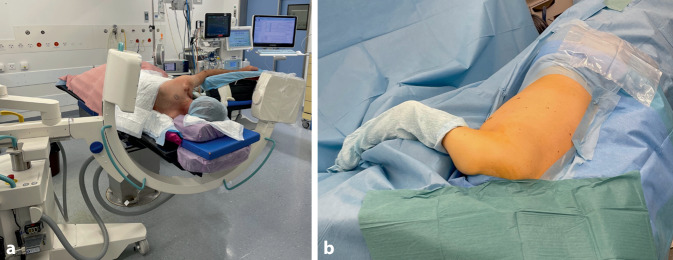
**a** Positioning of the patient and C‑arm. **b** Patient after draping

**Fig. 2:**
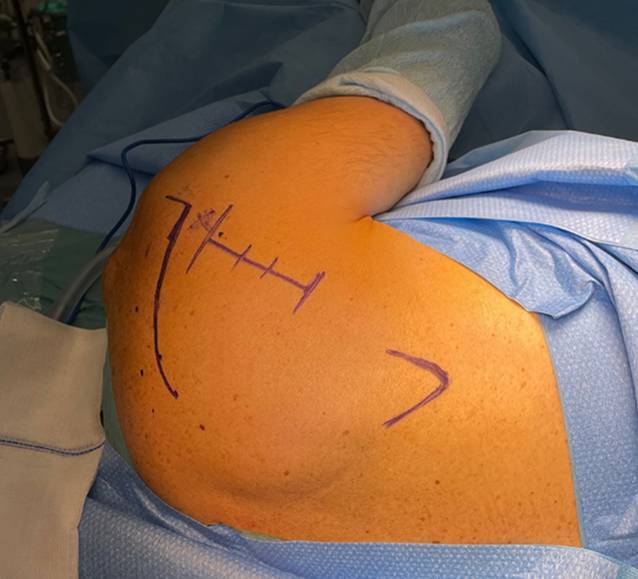
A straight incision along the lateral margin of the scapula or a slightly curved more cosmetic incision directed towards the axilla is performed. A full thickness skin and subcutaneous flap is created leaving the superficial deltoid fascia intact

- General anaesthesia with adequate muscle relaxation is mandatory
- Lateral position is preferred to prone. It allows for better control of arm position that can aid reduction and permits using the coracoid for the placement of an additional percutaneous joystick if needed to facilitate reduction in scapular neck fractures.
- Place the patient on the cantilevered end of a radiolucent operating table. Appropriate padding of the chest and the uninjured arm is necessary. The affected arm is draped free on a padded arm support or is held by a pneumatic arm holder allowing intraoperative limb positioning to aid reduction and imaging (Fig. 1).
- Fluoroscopy should be positioned perpendicular to the patient and table, entering from the cranial (or alternatively anterior/front) side of the patient. Preoperative trial imaging should be performed to confirm the ability to achieve a proper scapular Y view and an anteroposterior (AP) view of the shoulder (Fig. 2).

## Surgical technique

**Fig. 3:**
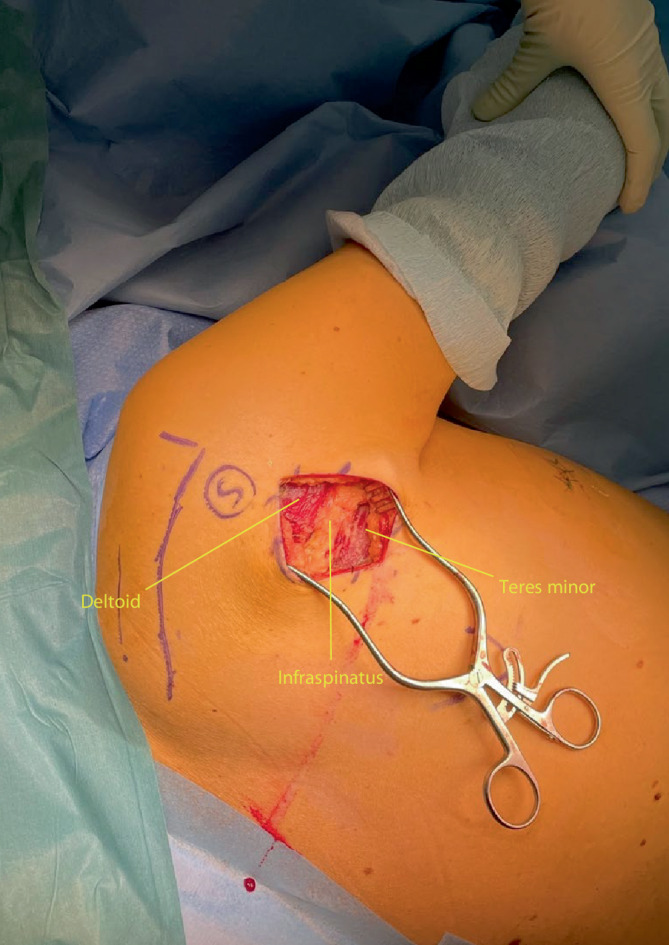
The deltoid margin, infraspinatus and teres minor are identified

**Fig. 4:**
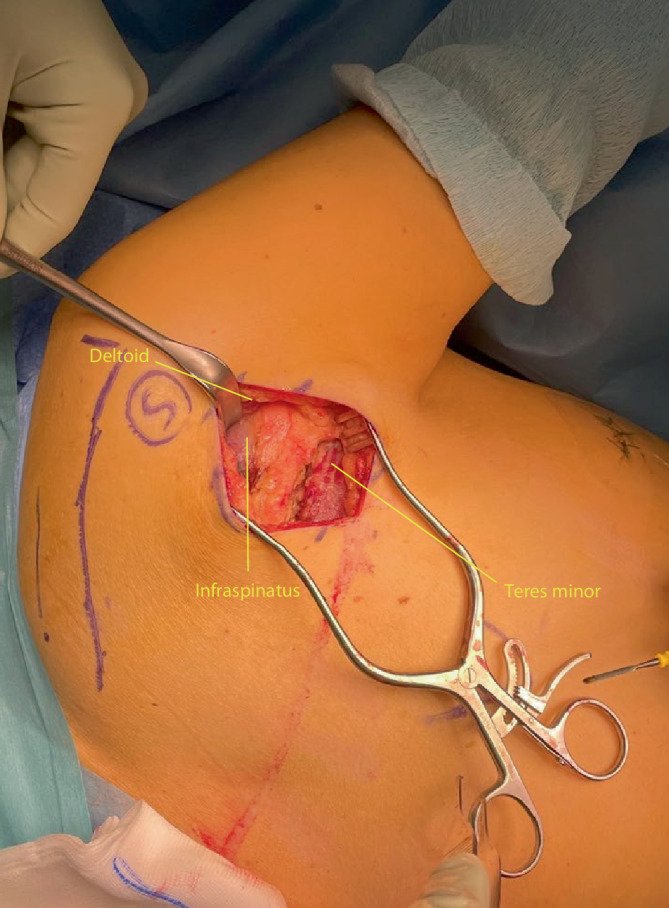
The deltoid is retracted cranially (abduction of the shoulder with additional padding may also help removing the deltoid muscle out of the operating field) and the interval between teres minor and infraspinatus is identified. This interval is frequently not well defined. A helpful anatomical landmark is the fact that the infraspinatus muscle usually has diamond shaped muscle fibres, whilst the teres minor has linear fibres. Furthermore the interval can also be found by palpating the lateral border of the scapula: infraspinatus lies superior and the teres minor inferior to the lateral scapular border. Failure to identify the correct interval might cause damage to the axial nerve that runs between the teres minor and major laterally

**Fig. 5:**
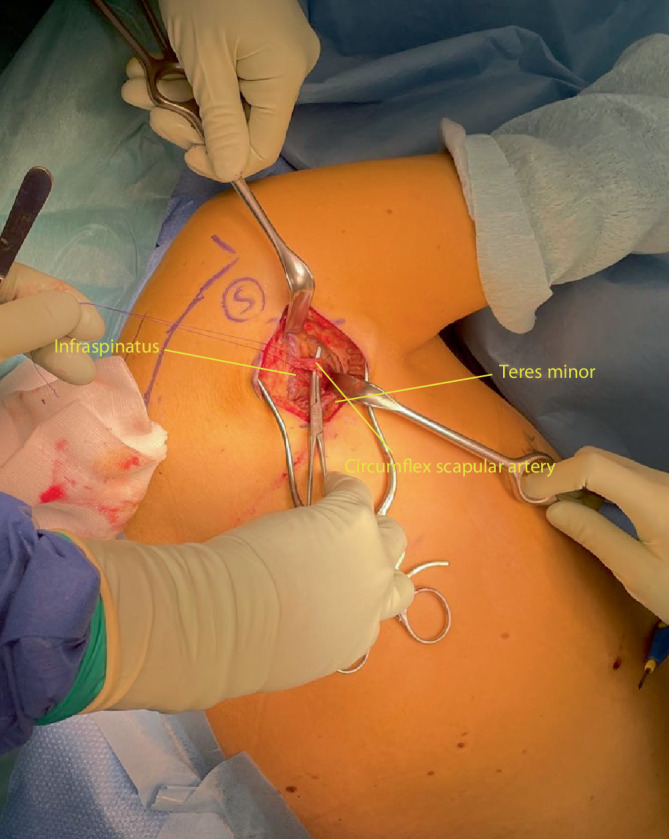
The ascending branch of the circumflex scapular artery frequently runs across the lateral margin of the scapula in the interval between teres minor and infraspinatus. This artery should be ligated or cauterized to prevent troublesome bleeding

**Fig. 6:**
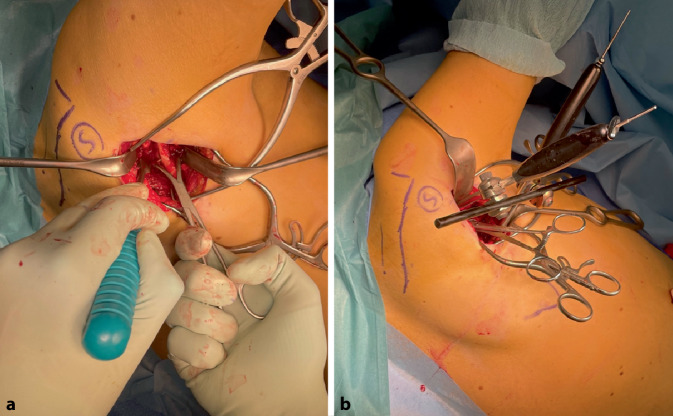
**a** After identification of the interval between the infraspinatus and teres minor the lateral margin of the scapula is identified. The fracture zone is exposed and cleared. **b** Reduction and alignment of the lateral margin is achieved using two reduction forceps on either side of the fracture. To maintain reduction, a 2.0 mm (or 2.7 mm) plate may be positioned and fixed on the lateral edge of the scapula (leaving space for a 2.7 mm plate on the surface of the scapular body which will ultimately provide the required stability). An alternative for maintaining reduction is the use of two Schanz screws (2.5 mm) and connecting them with a rod from the mini external fixator set

**Fig. 7:**
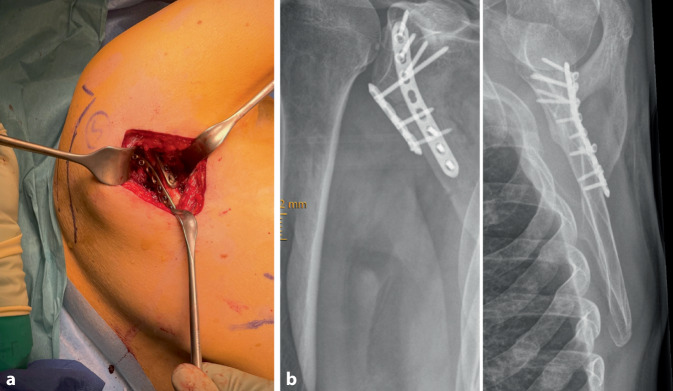
**a** The 2.7 mm plate should be precontoured keeping the glenopolar angle (30–40°) in mind. The plate is positioned on the dorsal aspect, along the lateral border of the scapula. Preliminary fixation with K‑wires is advisable. **b** It is important in this phase to obtain adequate intraoperative radiographs with a free projection of the joint glenohumeral joint to exclude intra-articular positioning of the most cranial K‑wire. The direction of the cranial K‑wire may be used as a reference when placing the screws in the cranial part (near the glenohumeral joint) of the plate. At the end of the procedure multidirectional intra-operative radiographs should be obtained

(Figs. 2, 3, 4, 5, 6 and 7)

## Special surgical considerations

(Fig. 8)

**Fig. 8:**
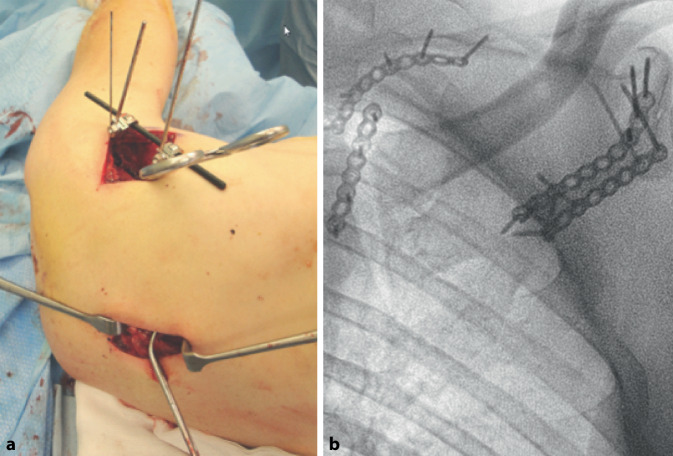
**a** In case of a comminuted lateral border in which rigid fixation is not possible or medial displacement (> 1 cm), an additional incision over the medial margin is performed. The medial incision and precedent reduction of the the margo medialis is made neutralizing the medial–lateral displacement of the scapular body fragment and adding reduction. Addressing the medial side first might also be beneficial in cases of severe comminution of the lateral margin where no good reference for reduction can be obtained. If required, additional windows can be made over the spina scapulae, in case of fractures extending to this part of the scapula. **b** The intra-operative images show fracture reduction using four 2.7 mm plates

### Postoperative management

Direct postoperative radiographs are recommended to assure an adequately reduced fracture and correct implant position. A sling is provided for comfort, pain control and soft tissue healing. Under supervision of a physiotherapist, assisted thoraco-scapular mobilization and range of motion exercises respecting pain perception are allowed. Resistance training and weight bearing will be allowed after the first outpatient follow-up after 6 weeks. Expected return to work ranges from 2 weeks, in case of desk job, but up to 3 months in case of physically demanding work.

### Errors, hazards, complications

- Screw perforation into the glenohumeral joint
- Damage to the supraclavicular nerve running through the spinoglenoid notch caused by either traction or a malpositioned Hohmann retractor
- Damage to the axillary nerve running through the interval between teres major and minor

## Results

### Outcome institution and literature

**Fig. 9:**
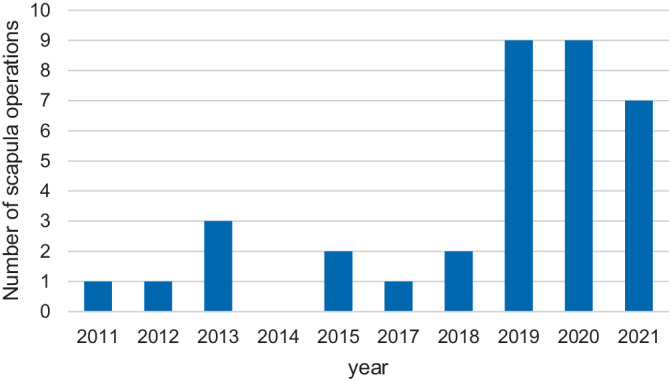
Number of osteosyntheses for scapula fractures on a yearly basis

We collected data of 35 patients treated with minimally invasive plate osteosynthesis (MIPO) between 2011 and 2021. The median follow-up was 8 months (range 3–31 months). All patients sustained high energy trauma (injury severity score > 16) and were predominantly male (only 1 female). Average age was 53 ± 15.1 years (range 21–71); 17 had a type B and 18 type F fracture according the AO/OTA classification. All patients suffered concomitant injuries of which thoracic (*n* = 33) and upper extremity (*n* = 25) injuries were most common. Average time to surgery was 6 ± 4 days (0–17 days; Fig. 9).

Predominantly double plating (*n* = 30) was performed using either a 2.0 mm or 2.7 mm plate to maintain reduction and a larger 2.7 mm plate (or 3.5 mm reconstruction plate in the period prior to 2019) for stability. Average time to union was 21.5 ± 8.5 (range 11–36) weeks. One patient underwent an additional osteosynthesis 3 months after initial surgery due to pain and lack of radiological signs of healing of a fracture extension into the spina scapulae. In the same patient the plate on the spina scapula was later removed due to plate irritation. In 2 patients postoperative images showed a screw protruding into the glenohumeral joint requiring revision surgery. After standardisation of intra-operative imaging following these 2 cases, intra-articular screw placement did not occur anymore. No patient developed an infection or sustained iatrogenic nerve injury.

The range of motion at the last outpatient follow-up visit was as follows: anteflexion median 165 (range 130–170), abduction median 165 (85–180), exo 60 (30–90), endo 80 (45–90). Median disabilities of arm, shoulder and hand questionnaire (DASH) score was 11 (range 5–31).

To the best of our knowledge two studies investigated outcomes specifically for minimally invasive plate fixation for scapular fractures [3, 4]. Gauger et al. published their retrospective case series of 7 male patients with a mean age of 39 years (range 19–75 years) and follow-up of a minimum 12 months and reported comparable results to our study [3]. The mean DASH score was 8.1 (range 0–52). Strength and motion returned to equivalency with the uninjured shoulder. The retrospective cohort study of Mannambeth et al. included 11 patients (10 men) with a mean age of 45 years (range 24–67 years) and follow-up of 16 months (range 12–28 months) [4]. They also demonstrated similar outcomes: The mean DASH score was 11.4 (range 0–51). Almost all patients returned to work after an average of 3.3 months (range 2 weeks–8 months). One patient did not because of concomitant brachial plexus injury. All fractures healed in their cohort without infections or nerve injury.

## Declarations

For this article no studies with human participants or animals were performed by any of the authors. All studies mentioned were in accordance with the ethical standards indicated in each case.
